# *In-situ* Observation of Cross-Sectional Microstructural Changes and Stress Distributions in Fracturing TiN Thin Film during Nanoindentation

**DOI:** 10.1038/srep22670

**Published:** 2016-03-07

**Authors:** Angelika Zeilinger, Juraj Todt, Christina Krywka, Martin Müller, Werner Ecker, Bernhard Sartory, Michael Meindlhumer, Mario Stefenelli, Rostislav Daniel, Christian Mitterer, Jozef Keckes

**Affiliations:** 1 Materials Center Leoben Forschung GmbH, Leoben, Austria; 2 Department of Materials Physics, Montanuniversität Leoben, Austria; 3 Ruprecht Haensel Laboratory, University of Kiel, Germany; 4 Helmholtz Zentrum Geesthacht, Geesthacht, Germany; 5 Department of Physical Metallurgy and Materials Testing, Montanuniversität Leoben, Austria

## Abstract

Load-displacement curves measured during indentation experiments on thin films depend on non-homogeneous intrinsic film microstructure and residual stress gradients as well as on their changes during indenter penetration into the material. To date, microstructural changes and local stress concentrations resulting in plastic deformation and fracture were quantified exclusively using numerical models which suffer from poor knowledge of size dependent material properties and the unknown intrinsic gradients. Here, we report the first *in-situ* characterization of *microstructural changes* and *multi-axial stress distributions* in a wedge-indented 9 μm thick nanocrystalline TiN film volume performed using synchrotron cross-sectional X-ray nanodiffraction. During the indentation, needle-like TiN crystallites are tilted up to 15 degrees away from the indenter axis in the imprint area and strongly anisotropic diffraction peak broadening indicates strain variation within the X-ray nanoprobe caused by gradients of giant compressive stresses. The morphology of the multiaxial stress distributions with local concentrations up to −16.5 GPa correlate well with the observed fracture modes. The crack growth is influenced decisively by the film microstructure, especially by the micro- and nano-scopic interfaces. This novel experimental approach offers the capability to interpret indentation response and indenter imprint morphology of small graded nanostructured features.

In materials science, the synthesis of novel polycrystalline materials with enhanced mechanical properties, such as fracture toughness and ultimate tensile strength, strongly depends on the understanding of microstructural changes and internal stress distributions accompanying plastic deformation and resulting in fracture^1^,^2^,^3^,^4^. Local strain characterization during material deformation has been experimentally achieved using X-ray diffraction (XRD) in polycrystalline coarse-grained metals with a spatial resolution in the μm range^5^,^6^. In mechanically loaded small features like thin films with sizes in the μm range or even smaller, it is difficult to experimentally determine three-dimensional stress distributions due to dimensional and geometrical constraints. For this reason, indentation-induced stress evolution has been assessed exclusively by numerical models^7^,^8^, which however suffer from the poor knowledge of size-dependent material parameters^1^ and intrinsic gradients of microstructure and residual stresses^9^,^10^ typically present in nanomaterials. Traditionally those models have also been used to interpret the indentation response of thin films represented by integral values of hardness and load-deflection curves^11^,^12^,^13^.

Protective hard nanocrystalline thin films like TiN, CrN and TiAlN represent a material class whose mechanical properties such as hardness and toughness are commonly determined by indentation tests^10^,^14^. There have been a few experimental *ex-situ* and *in-situ* transmission electron microscopy (TEM) studies, which revealed microstructural changes after and during indentation like intergranular sliding of V-shaped grains^10^, dislocation motion^15^ and crack propagation in monolithic as well as multi-layered thin films^16^. A recent report by Li *et al.*^14^ has documented that the indentation response of metal-ceramic films is strongly size-dependent. For instance, the cracking observed in 50 nm thick TiN layers was suppressed in 5 nm thick layers confined between two Al layers giving rise to Al-TiN plasticity.

Though the indentation-induced microstructural changes have been extensively studied primarily using *ex-situ* and *in-situ* TEM^14^,^15^,^16^,^17^,^10^, *in-situ* experimental characterization of internal stress distributions and microstructural changes occurring in *thin film volume* under and around the contact area during the indenter tip penetration have remained experimentally inaccessible. Consequently, magnitudes of anisotropic stress concentrations resulting in various indentation failure modes are unknown. This is also the reason why the optimization of thin film mechanical properties is in practice performed by tedious trial-and-error variations of film composition, residual stress gradients and microstructure.

This work presents a pioneering experimental *in-situ* X-ray nanodiffraction approach, which is used to resolve simultaneously microstructural changes and stress distributions in a TiN thin film during step-wise indentation by analysing the diffraction signal from film crystallites. The main aims of the experiment are (i) to analyse cross-sectional microstructural changes and (ii) to quantify magnitudes of anisotropic stress concentrations around the indenter contact area. Complementary, a finite element (FE) model is built and *in-situ* indentation as well as micro-cantilever bending experiments are performed in a scanning electron microscope (SEM) in order to better interpret the deformation and fracture modes within the film.

## Introduction

### *In-Situ* Synchrotron Experiment and Sample Nature

**Figure 1:**
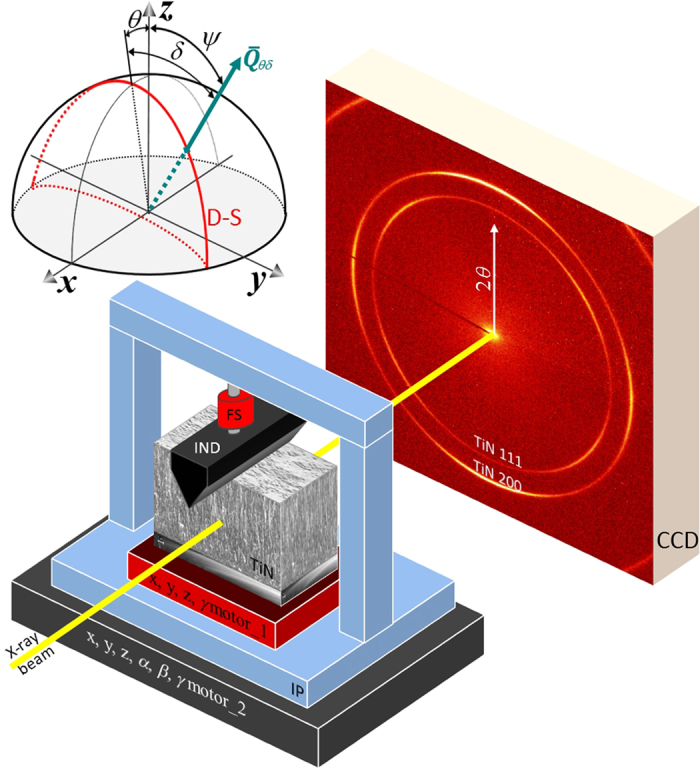
A schematic setup of the *in-situ* X-ray nanodiffraction experiment. A 9 μm thick TiN film on a steel substrate is loaded against a diamond wedge indenter (IND) using motor_1 along the *z* axis, accompanied by a simultaneous measurement of the applied load by the force sensor (FS). Motor_2 is used to move the indenter platform (IP) with the sample in the beam along *y* and *z* directions. The 2D charge-coupled device (CCD) detector collects TiN Debye-Scherrer rings for each *y* and *z* cross-sectional sample position. The stereographic projection above the indenter shows schematically a projection of a Debye-Scherrer ring (D-S), a representative orientation of the diffraction vector 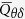, whose orientation in the sample coordinate system *x*, *y* and *z* is defined by angles δ, θ and ψ. The angle θ represents Bragg’s angle, angle δ represents azimuthal position of 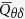 along D-S and ψ is the angle between the sample normal and the diffraction vector.

The *in-situ* experimental setup was developed at the Nanofocus Endstation of MiNaXS (P03) beamline^18^ at the PETRA III synchrotron radiation source in Hamburg, Germany (Fig. 1). The monochromatic X-ray beam with a photon energy of 14.73 keV is focused by a pair of elliptical mirrors in crossed geometry providing the spatial resolution of 200 × 200 nm^2^. A cross-sectional sample lamella consisting of the substrate and the film with a thickness of 40 μm (in the beam direction) is analysed in transmission in wide angle diffraction geometry (Fig. 1). The sample is integrated into a home-built indenter, which is equipped with a 3 N strain gauge force sensor and with a variety of piezo positioners for the sample movement. During the *in-situ* XRD experiment, the whole indenter system with the loaded sample is moved with an accuracy below 100 nm in a grid perpendicular to the beam direction and diffracted photons are collected using a 2D detector (Fig. 1).

An important feature of the setup is the use of a wedge indenter, whose edge is oriented parallel to the beam direction, similar as in our previous *ex-situ* study^19^. In this way, the indentation geometry is significantly simplified because the primary X-ray beam oriented perpendicular to the film cross-section penetrates the sample always at constant *y* and *z* distances from the indenter wedge (Fig. 1). This implies that the strain magnitude in the irradiated volume is relatively homogeneous, as far as strain relaxations at the lamella borders and the strain variation along the *x* axis can be neglected. The approach allows for determining the volume-averaged 2D distributions of microstructure as well as strains across the film cross-section at individual indentation steps with the spatial resolution of the beam size.

**Figure 2:**
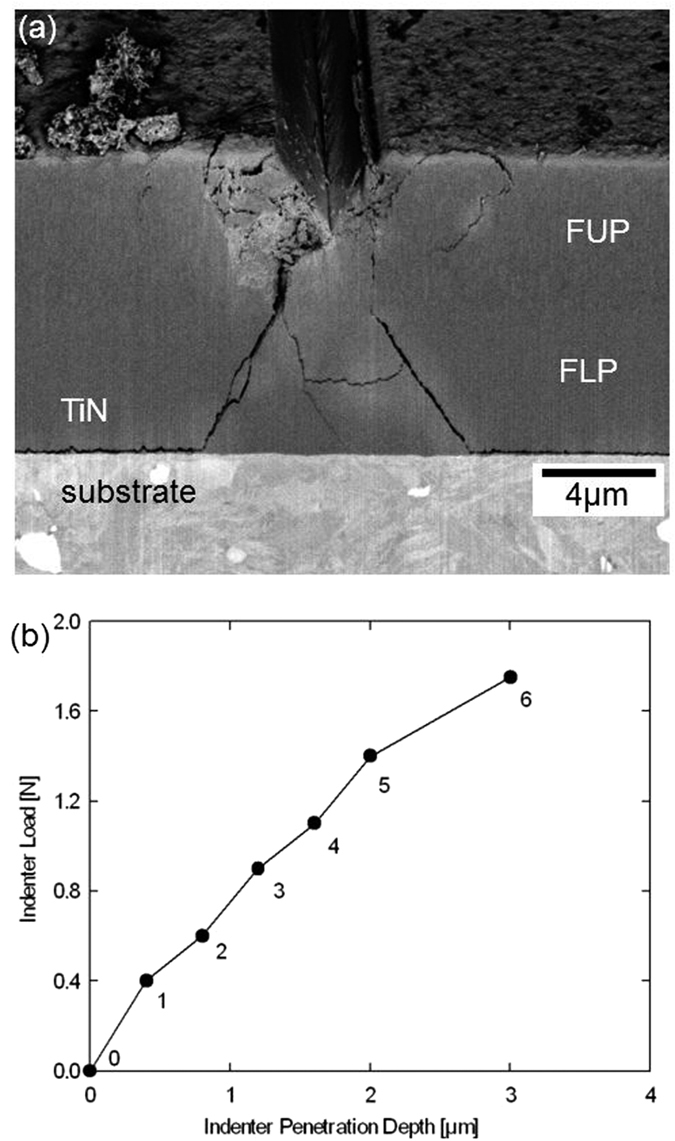
Results from the *in-situ* wedge indentation experiment. (**a**) A scanning electron microscopy micrograph of a TiN film cross-section on a steel substrate after the indentation at the synchrotron beamline with two dominant symmetric cracks proceeding perpendicular and at an angle of ~30 degrees towards the film-substrate interface in FUP and FLP, respectively. (**b**) The corresponding load-displacement curve indicates different stages of the indentation experiment.

**Figure 3:**
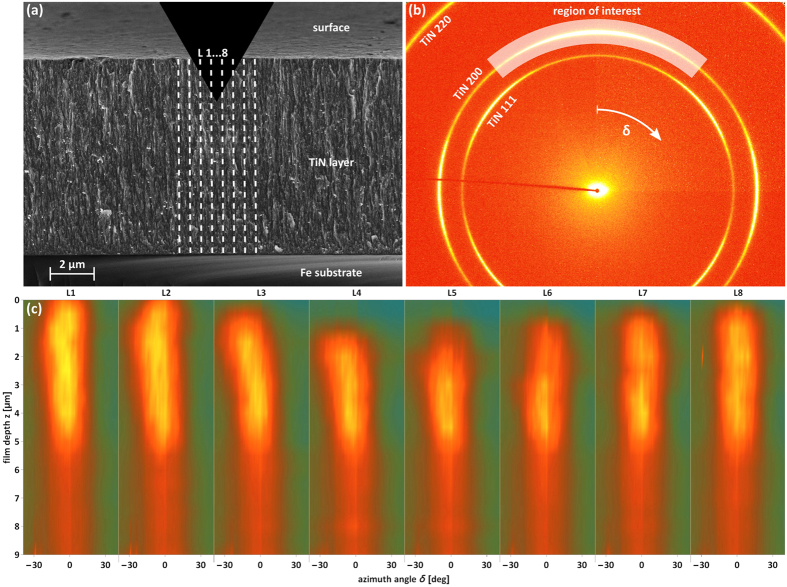
Local crystallographic texture changes during indentation with a load of 1.4N. (**a**) On a SEM micrograph of the film cross-section, L1-L8 indicates schematically the positions at which cross-sectional X-ray nanodiffraction scanning was performed. Also, the nanocrystalline nature of the film with needle-like grains can be recognized. (**b**) Azimuthal distributions of intensities *I*(δ) of TiN 200 Debye-Scherrer rings were evaluated in the region of interest. (**c**) The changes in *I*(δ) at different sample film depths and L1-L8 positions document that the cubic TiN needle-like crystallites were tilted left and right away from the wedge axis up to 15 deg. The widening of the azimuthal distributions *I*(δ) in the contact area (**c**) demonstrates an increase in the film mosaicity and indirectly a film densification as a result of the irreversible film deformation in the imprint area are visible in Fig. 2a.

The examined nanocrystalline 9 μm thick TiN film with columnar grain morphology (Figs 2a and 3a) was deposited on a steel substrate using an industrial-sized plasma-assisted chemical vapour deposition and nitriding plant. The central component is a hot-wall reactor featuring wall temperatures of up to 873 K. The reactor is supplied with the process gases (TiCl_4_, H_2_, N_2_, Ar) by a standard gas mixing system using mass flow controllers. The plasma is sustained by applying direct current pulses to the substrates. For the deposition, a pressure of 200 Pa was applied. The columnar grains were oriented perpendicular to the film/substrate interface. During the deposition, the substrate temperature was increased abruptly from 813 to 853 K after the growth of the first half of the film was completed. The temperature increase resulted in an increase of the TiN crystallite size and in the formation of a sharper TiN 100 fibre texture^20^. The film upper and lower parts deposited at different temperatures resulting in the formation of different microstructures and stress states *will be further denoted as FUP and FLP.*

## Results and Discussion

### Indention Imprint Morphology

In Fig. 2a, a representative cross-section of the film prepared by focused ion beam polishing after the *in-situ* synchrotron experiment demonstrates deformation modes accompanying the wedge indentation. The indentation results in the formation of a wedge imprint, which spans across the whole lamella width (along the *x* axis in Fig. 1). Left and right to the imprint, networks of cracks indicate that stress concentrations exceeded the material strength limit. Additionally, there are two dominant *primary cracks* starting at the imprint faces and progressing towards the interface. In FUP, the cracks are oriented perpendicular to the film/substrate interface whereas in FLP the cracks proceed at an angle of about 30 degrees with respect to the film normal. At the film/substrate interface, the primary cracks are deflected and propagate along the interface.

In Fig. 2b, a load versus penetration-depth dependence measured during the *in-situ* experiment is presented. At each indentation step from 0 to 6, the sample cross-section was analysed using XRD at up to 525 *y* and *z* positions. After the indenter load was increased to above 1.9 N, it dropped rapidly to 1.3N. The film cross-section in Fig. 2a was recorded after the film failure.

In order to better interpret the morphology of the *primary cracks* visible in Fig. 2a, (i) an *in-situ* indentation experiment on the TiN thin film and (ii) a bending experiment on a TiN micro-cantilever machined using a focused ion beam (FIB) were performed in SEM, *in addition* to the *in-situ* experiment carried out at the synchrotron beamline. Morphologies of the indenter imprint as well as the cantilever together with corresponding load-deflection curves are presented in supplementary Figs 1–4. Moreover supplementary Videos 1 and 2 document the indenter penetration into the film and the cantilever bending in SEM, respectively. In both SEM experiments, there are cracks initiated at the film surface which are deflected at the FUP/FLP interface, in agreement with the observation in Fig. 2a. These observations document the importance of the interface between both film regions in the mechanical response of the film. From the analysis of the SEM images (Fig. 2a, supplementary Figs 1 and 2), however, the reason for the crack deflection at the FUP/FLP interface is not obvious. Moreover, the SEM experiment on the cantilever documents that the crack initiated on the film surface propagates parallel to the elongated TiN columnar grains in FUP (as visible in supplementary Fig. 1c), in agreement with the primary crack behaviour from Fig. 2a. The crack morphology observed in FUP (Fig. 2a) *suggests* that the deformation behaviour is dominated by *fracture of inter-columnar grain boundaries*^21^, which is caused by relatively small cohesive energy of the grain boundaries between the needle-like TiN nanograins of high strength^22^. This is in agreement with our results from similar nanoceramic thin films with columnar grain microstructures, where also intergranular fracture was reported^23^,^24^. It also agrees well with the results from experiments on nanoceramic films reported by others^25^,^26^,^17^,^27^,^28^.

### Qualitative Texture Analysis

Some indentation-induced cross-sectional microstructural changes in the thin film volume can be understood from the analysis of the azimuthal morphology of Debye-Scherer rings. In Fig. 3, azimuthal distributions of the diffraction intensity along TiN 200 rings *I*(*δ*) collected from the film area around the indenter imprint are analysed. In the unloaded state, azimuthal maxima of TiN 200 rings were observed at *δ* = 0 degrees (as can be seen in Fig. 3b) documenting a relatively broad 100 fibre texture^20^,^29^. During the indentation, the positions of the azimuthal maxima in *I*(*δ*) dependencies shifted up to 15 degrees counter-clockwise and clockwise in the diffraction patterns collected left and right from the wedge axis, respectively. This is documented in Fig. 3c by results from eight cross-sectional line-scans measured under the indenter (along the *z* axis according Fig. 1) showing an azimuthal shift of the azimuthal maxima in *I*(*δ*) dependencies as a function of the film depth. This observation documents that the indentation of the film with columnar grain morphology resulted in the tilt of the crystallites under the wedge and thus caused a local change of the crystallographic texture and texture sharpness in FUP. The sample analysis after the *in-situ* experiment indicated that this texture change was partly irreversible.

### Peak Broadening Analysis

**Figure 4:**
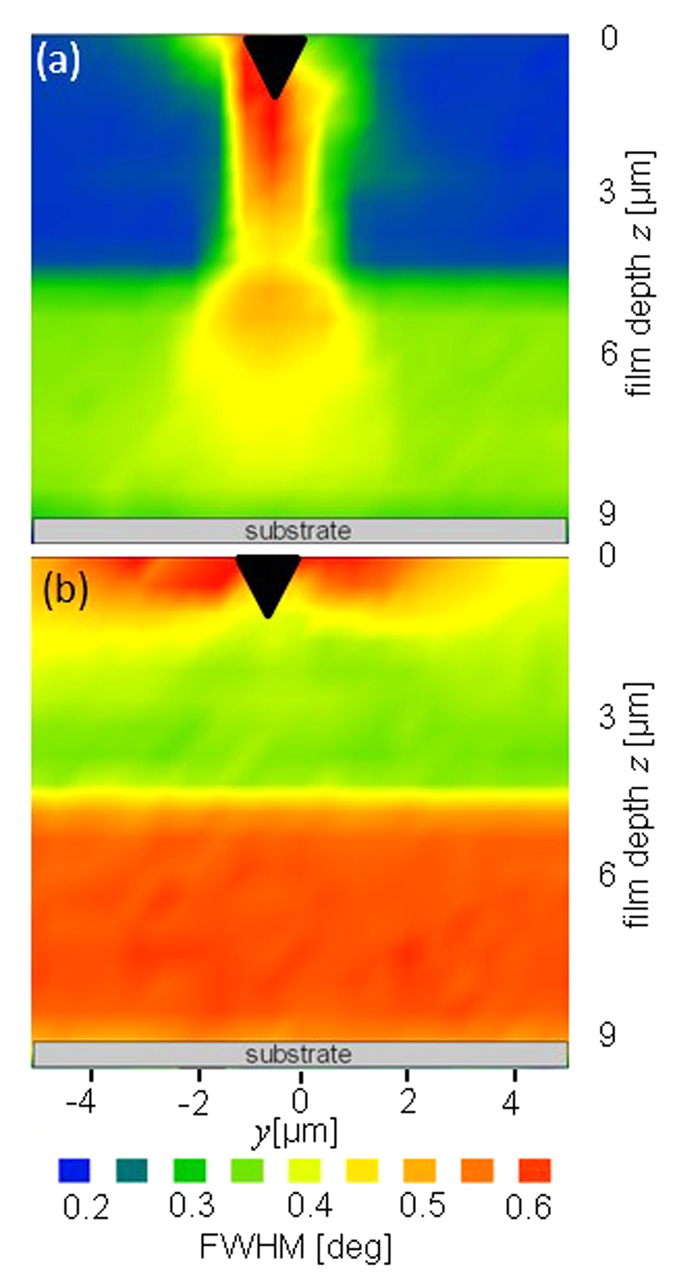
Cross-sectional distributions of TiN 200*FWHM*_*δ*_(*y*, *z*)observed during the indentation with a load of 1.4N. The local increase in out-of-plane *FWHM*_90_(*y*, *z*) (**a**) and in-plane *FWHM*_0_(*y*, *z*) (**b**) correlates well with the concentrations of out-of-plane and in-plane stress components *σ*_22_(*y*, *z*) and *σ*_33_(*y*, *z*) in Fig. 6. Different *FWHM*_90_(*y*, *z*) and *FWHM*_0_(*y*, *z*) in FUP and FLP regions demonstrate the presence of needle-like crystallite morphology as well as an abrupt change in the film microstructure at the FUP/FLP interface.

Another information on the film microstructural changes during the *in-situ* synchrotron indentation experiment can be obtained from the analysis of full width at half maximum *FWHM*_*δ*_ of TiN 200 Debye-Scherrer rings evaluated for different values of the azimuthal angle *δ* (defined in Figs 1 and 3b). In Fig. 4a,b, in-plane and out-of-plane thin film cross-sectional distributions of *FWHM*_*δ*_(*y*, *z*), *FWHM*_0_(*y*, *z*) and *FWHM*_90_(*y*, *z*), determined for TiN 200 azimuthal positions *δ*  = 0 and 90 degrees, respectively, and the film cross-sectional positions *y* and *z* are presented. The data exhibit a very significant increase in *FWHM*_*δ*_(*y*, *z*) under the indenter in Fig. 4a and next to the indenter in Fig. 4b. This behaviour could be attributed (i) to the plastic deformation in the nanoceramic film resulting in the formation of crystallographic defects within TiN grains and/or (ii) to microscopic strain gradients within the irradiated volume. In the case of plastic deformation, however, a broadening of practically all TiN *hkl* reflections (except those on which the gliding takes place) could be expected^30^. Since the *FWHM*_*δ*_(*y*, *z*) increases in Fig. 4a,b occur only for *δ* ≈ 0 and *δ* ≈ 90 degrees, it can be supposed that the main reason for this phenomenon is a micro-scale variation of some stress distributions in the fracturing film next to the indenter (Fig. 4a) and under the indenter (Fig. 4b).

A comparison of *FWHM*_*δ*_(*y*, *z*) values from FUP and FLP in Fig. 4 indicates (i) an abrupt change in *FWHM*_*δ*_(*y*, *z*) at the boundary between FUP and FLP and (ii) larger *FWHM*_90_(*y*, *z*) values in Fig. 4b compared to *FWHM*_0_(*y*, *z*) in Fig. 4a in the respective areas not affected by the indentation. It is known that *FWHM*_*δ*_(*y*, *z*) depends on the size of coherently diffracting domains and/or on the magnitude of strains of the second and the third order in crystallites. The relatively large *FWHM*_90_(*y*, *z*) observed in Fig. 4b indicates diffraction on crystallographic planes oriented perpendicular to the film-substrate interface (*i.e.* diffraction vectors 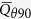 are oriented parallel to the interface, *cf*. Fig. 1)^29^. Since the in-plane thickness of TiN film needle-like crystallites is smaller than their out-of-plane length, *FWHM*_90_(*y*, *z*) are consequently significantly larger than *FWHM*_0_(*y*, *z*)^29^. Additionally, the differences in *FWHM*_*δ*_(*y*, *z*) between FUP and FLP document that the crystallites in FUP are larger and/or possess less microstructural defects, since higher deposition temperature was applied during FUP growth.

### Cross-Sectional Stress Analysis

**Figure 5:**
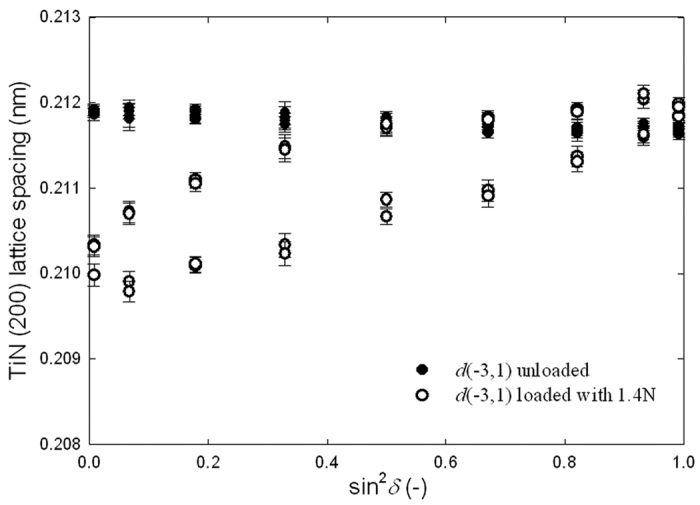
Development of the TiN (200) lattice spacing for various orientations of the diffraction vector 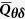 and for cross-sectional sample positions y = −3 and z = 1 μm. The filled and open points represent *σ*_11_(−3, 1) data from unloaded and sample loaded with 1.4N force. The split dependencies from the loaded sample indicate a presence of *σ*_23_(*y*, *z*). Cross-sectional 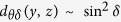
dependencies are available as supplementary Figs 5 and 6.

The origins of the fracture modes visible in Fig. 2a as well as the localised *FWHM*_*δ*_(*y*, *z*)increases in Fig. 4 can be understood from the analysis of cross-sectional multi-axial stress distributions accompanying the wedge indentation. Debye-Scherrer rings collected using the 2D detector at the thin film cross-sectional position *y* and *z* (Fig. 1) were used to evaluate the lattice spacing *d*_*θδ*_(*y*, *z*) of TiN (200) crystallographic planes as a function of the ring azimuthal angle *δ* using Bragg’s law by analysing diffraction angle 2*θ* azimuthal dependencies 2*θ*(*δ*) of TiN 200 reflections. For this reason, every collected 2D pattern was treated using the software Fit2D^31^ and *d*_*θδ*_(*y*, *z*) values were determined for 36 azimuthal angle δ sections. Every *d*_*θδ*_(*y*, *z*) value represents an X-ray probe volume-averaged lattice parameter for a diffraction vector 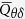 orientation defined by the angles δ and θ in Fig. 1. In Fig. 5, 36 dependencies *d*_*θδ*_(*y*, *z*) on sin^2^*δ* are presented for unloaded sample and sample loaded with 1.4N for *y* = −3 and *z* = 1 μm. In supplementary Figs 5 and 6, cross- sectional dependencies of lattice parameters *d*_*θδ*_(*y*, *z*) as a function sin^2^*δ* are shown for the unloaded film and the film loaded with 1.4N. Whereas in the unloaded sample all *d*_*θδ*_(*y*, *z*) ~ sin^2^*δ* dependencies were linear, there was a significant occurrence of regions with split dependencies *d*_*θδ*_(*y*, *z*) ~ sin^2^*δ* in the loaded film. This effect documents the presence of shear stresses in respective regions of the loaded film^32^.

The unstressed lattice parameter *d*_*o*_ = 0.21188 nm was determined from diffraction data collected from the surface region of the unloaded film by considering the sample stress free direction^33^, an equibiaxial stress state and TiN elastic constants^34^,^35^. By comparing *d*_*θδ*_(*y*, *z*) and *d*_*o*_, X-ray elastic strain *ε*_*θδ*_(*y*, *z*) for the diffraction vector 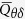 orientation specified by the angles δ and θ (Fig. 1) and for the cross-sectional thin film position *y* and *z* was calculated as


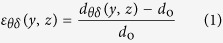


The measured strain *ε*_*θδ*_(*y*, *z*) can be expressed as a function of unknown strain components *ε*_*ij*_(*y*, *z*) defined in the (sample) coordinate system with axes *x, y* and *z* from Fig. 1 as follows^36^


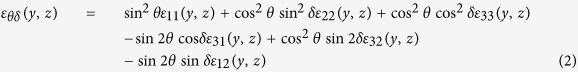


Similarly, the measured strain *ε*_*θδ*_(*y*, *z*) can be expressed as a function of unknown stress components *σ*_*ij*_(*y*, *z*)defined in the sample coordinate system as follows


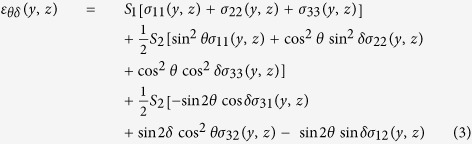


The parameters 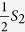
and *S*_1_in Eq. 3 are X-ray elastic constants of the film with 100 fibre texture calculated for the TiN 200 reflection^20^,^29^,^35^.

In the case of the unloaded sample, it was supposed that the stress state in the film was triaxial with non-zero principal stress components *σ*_*ii*_(*y*, z) ≠ 0, whereas shear stress components *σ*_12_(*y*, *z*) and *σ*_13_(*y*, *z*) were neglected for simplicity. Moreover, laboratory as well as synchrotron cross-sectional characterization indicated that the film in-plane stress was equi-biaxial with *σ*_11_(*y*, z) ≅ *σ*_22_(*y*, z). Therefore cross-sectional *residual stress distributions* in the *unloaded sample* were evaluated using


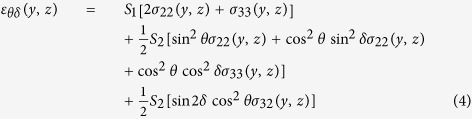


A system of linear equations based on Eq. 4 was built. On the left hand side, 36 measured values of strains *ε*_*θδ*_(*y*, *z*) evaluated from one Debye-Scherer ring (like those in Fig. 5 and in supplementary Fig. 5) were applied whereby the unknown parameters *σ*_22_*(y*, *z*), *σ*_23_*(y*, *z*), and *σ*_33_*(y*, *z*) from Eq. 4 right hand side were fitted to the measured data using least-squares refinement.

In the loaded sample, due to the specific experiment geometry, it was supposed that the pressure induced by the indenter caused the following changes in the film stress state:

1. due to the experiment geometry and the wedge shape, stress components *σ*_22_(*y*, *z*), *σ*_33_(*y*, *z*) and *σ*_23_(*y*, *z*) were modified during indentation.

2. shear stress components *σ*_13_(*y*, *z*) and *σ*_12_(*y*, *z*) remained relatively small in the loaded sample and were neglected.

3. the magnitude of the in plane stress component *σ*_11_(*y*, *z*) did not change significantly during loading compared to the unloaded sample. The main arguments for this assumption are (i) the firm thin film fixing to the substrate and (ii) an absence of a force acting along the film *x* direction (Fig. 1) during the indentation experiment. Therefore cross-sectional residual stress distributions in the *loaded sample* were evaluated using


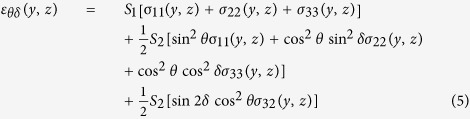


Also in this case a system of linear equations based on Eq. 5 was built. For every measured *y* and *z* sample position, 36 measured values of strains *ε*_*θδ*_(*y*, *z*) were applied whereby the unknown parameters *σ*_22_(*y*, *z*), *σ*_23_(*y*, *z*) and *σ*_33_(*y*, *z*) from Eq. 5 were fitted to the measured data using least-squares refinement. The values *σ*_11_(*y*, *z*) in Eq. 5 are identical with residual stress data in the unloaded sample and therefore do not represent variables in Eq. 5. The stress magnitudes in unloaded and loaded sample were evaluated with a numerical error smaller than 15%.

**Figure 6:**
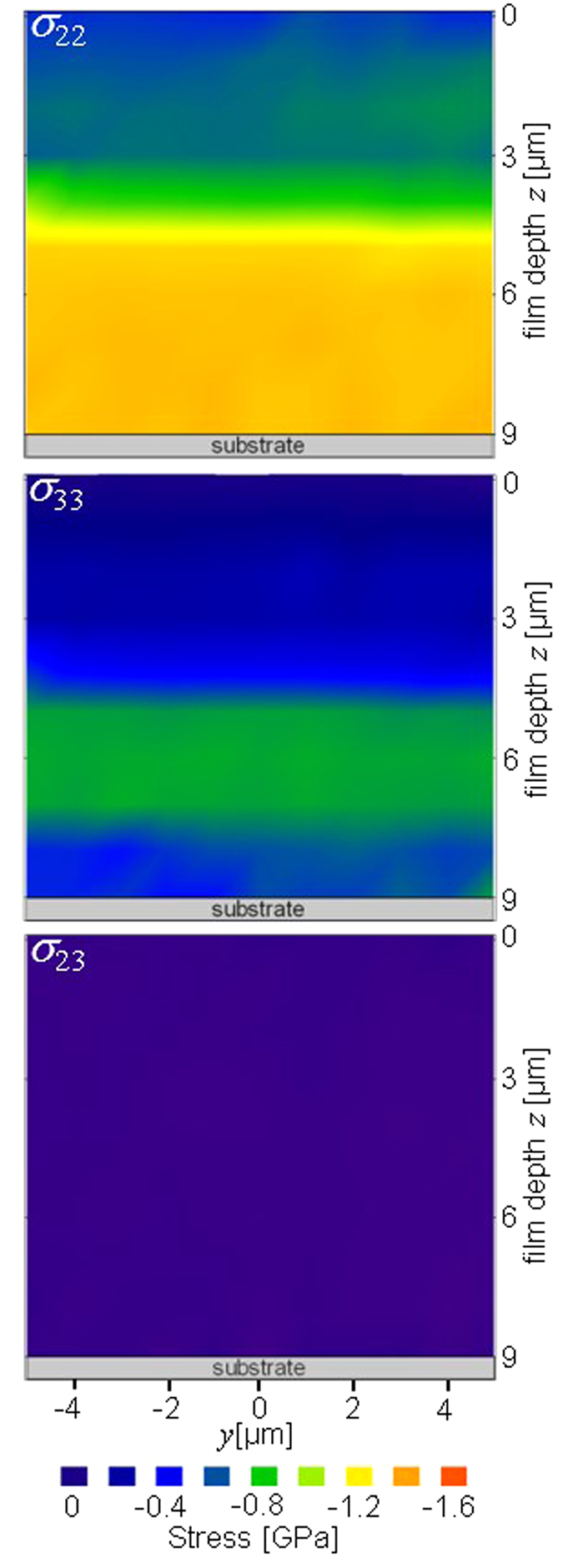
Residual stress distributions *σ*_22_(*y*, *z*), *σ*_23_(*y*, *z*) and *σ*_33_(*y*, *z*) at the positions *y*, *z* in the as-deposited 9 μm thick TiN film. The residual stress values demonstrate the presence of two film regions FUP and FLP with different microstructure as indicated by *FWHM*_δ_(*y*, *z*) in Fig. 4.

**Figure 7:**
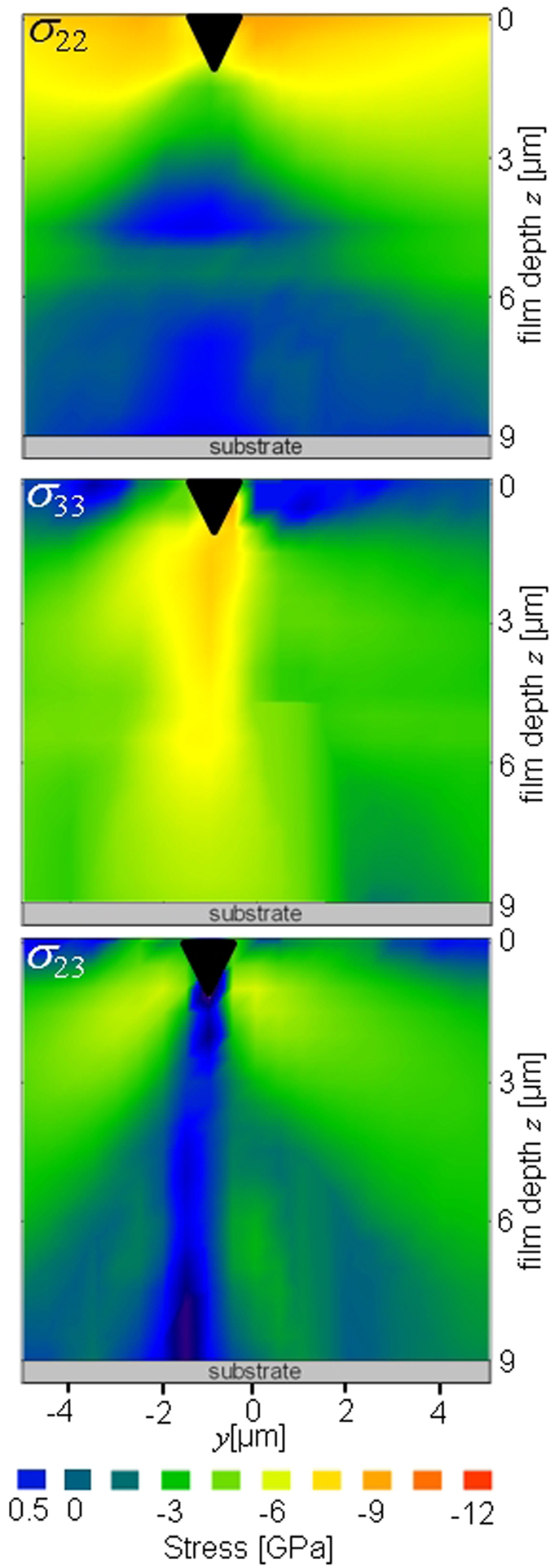
Experimental stress distributions *σ*_22_(*y*, *z*), *σ*_23_(*y*, *z*) and *σ*_33_(*y*, *z*) at the positions *y*, *z* in the 9 μm thick TiN film loaded with 1.4 N. The stress concentrations and distributions can be correlated with fracture modes visible in Fig. 2a as well as with the *FWHM*_*δ*_(*y*, *z*) values in Fig. 4. Due to presentation reasons, the actual magnitude of the shear stresses *σ*_33_(*y*, *z*) is shown as −|*σ*_33_(*y*, *z*)|.

Cross-sectional distributions of stresses *σ*_22_(*y*, *z*), *σ*_23_(*y*, *z*) and *σ*_33_(*y*, *z*) evaluated using Eqs 4 and 5 in the unloaded sample and in the TiN thin film loaded with 1.4 N are presented in Figs 6 and 7, respectively.

Before the indentation, the as-deposited film possessed an in-plane compressive stress *σ*_22_(*y*, *z*) in the range from −0.4 to −1.4 GPa (Fig. 6). The higher compressive in-plane stresses in FLP can be interpreted by a higher density of grain boundaries and microstructural defects^37^ in FLP as indicated also by an increase in *FWHM*_*δ*_(*y*, *z*) of TiN reflections from this region in Fig. 4. The proportionality between the magnitude of in-plane compressive stresses and nano-grain microstructure with small crystallites and high density of grain boundaries is a typical feature of nanoceramic thin films and has been extensively discussed in our previous reports^38^,^39^,^37^. The maximum value of the out-of-plane component *σ*_33_(*y*, *z*) of −0.8 GPa was observed at a depth of ~5–7 μm (in Fig. 6) whereas above and below this region *σ*_33_(*y*, *z*) stresses were significantly smaller or negligible. The presence of non-zero *σ*_33_(*y*, *z*) stresses in the film can be interpreted by the presence of two sublayers with different microstructure in the film. Since crystallite size was small and grain boundary density large in FLP, as documented by larger *FWHM*_*δ*_(*y*, *z*) from this regions (in Fig. 4), the subsequent growth of FUP sublayer at higher deposition temperature resulted in the formation of out-of-plane stresses in FLP^9^. It can be supposed that FUP sublayer hindered the out of plane stress relaxation of FLP sublayer. The shear stress component *σ*_23_(*y*, *z*) was found to be smaller than −0.1 GPa across the whole film thickness in the unloaded film (Fig. 6) as can be documented also by an absence of splitting of *d*_*θδ*_(*y*, *z*) ~ sin^2^*δ* dependencies in supplementary Fig. 5.

The cross-sectional stress distributions *σ*_22_(*y*, *z*), *σ*_23_(*y*, *z*) and *σ*_33_(*y*, *z*) inside the film under the constant indenter load of 1.4 N are presented in Fig. 7. A very high in-plane stress *σ*_22_(*y*, *z*) up to −11 GPa close to the faces of the indenter in the near-surface area can be attributed to the in-plane elastic deformation caused by the wedge volume being pressed into the film. The compressive in-plane stresses decrease as a function of the in-plane distance from the wedge edges. The maximal *σ*_22_(*y*, *z*) value of −16.5 GPa was observed next to the indenter edge in the film loaded with 1.75 N. The *σ*_22_(*y*, *z*) stress distribution in Fig. 7 with high in-plane near-surface stresses correlates well with the film near-surface fracture network visible in Fig. 2a next to the imprint. This correlation strongly suggests that the main reason for the near-surface film fracturing next to the wedge faces was the high magnitude of compressive in-plane stress *σ*_22_(*y*, *z*), which was beyond the material fracture limit. A comparison of *σ*_22_(*y*, *z*) and *FWHM*_90_(*y*, *z*) distributions in Figs 7 and 4b shows that the increase in peak width correlates with the high *σ*_22_(*y*, *z*) concentrations in the film near-surface region.

Directly under the indenter tip, the formation of in-plane tensile stresses *σ*_22_(*y*, *z*) up to about 500 MPa was observed due to the in-plane film elongation induced by the indenter being pressed into the film (Fig. 7). Since fracture strength up to few GPa was reported for nanocrystalline TiN thin films with columnar grain morphology^40^,^41^, it can be supposed that the tensile stresses below 1 GPa observed in FUP contributed to the formation of two primary cracks (Fig. 2a) but did not represent the main driving force for the intergranular fracture.

The region with tensile in-plane stresses under the indenter in Fig. 7 is interrupted by a narrow zone with compressive stresses located in FLP area next to FUP/FLP interface. This narrow zone with in-plane compressive stresses in Fig. 7 is a result of the remaining compressive residual state formed during the films synthesis and visible in Fig. 6. As Fig. 2a shows, the two primary cracks proceeding along the grain boundaries in FUP are deflected at the interface between FUP/FLP and continue to grow at an angle of about 30 degrees with respect to the sample film normal towards the film-substrate interface. One can expect that the deflection was caused (i) by this localised in-plane compressive stress zone present in FLP (Fig. 7) and/or (ii) by the abrupt microstructural change manifested by the abrupt change in *FWHM*_*δ*_(*y*, *z*) at the FUP/FLP interface (visible in Fig. 4). Larger *FWHM*_*δ*_(*y*, *z*) magnitudes recorded from FLP compared to those from FUP (*cf.* Fig. 4) indicate that the crystallites in FLP are smaller and/or possess a higher density of point defects. Thus, also the different nature of the TiN nanocrystalline grains in FLP could contribute to the crack deflection. The question arises here, whether the fracture mode in FLP was still intergranular or if the two cracks grew also across the nanocrystalline grains.

The two primary cracks located at the film/substrate interface (visible in Fig. 2a) can be attributed to the presence of tensile stress distributions *σ*_22_(*y*, *z*) at the interface (up to about 300 MPa) visible in Fig. 7. The tensile stresses at the interface were formed as a result of the localized in-plane elongation of the film, as well as substrate bending during the indenter penetration into the material. The crack growth along the interface can be classified also as an adhesion failure. The reason why the two primary cracks grew only outward from the imprint center and that there was a region with a mechanically stable film/substrate interface could be partly interpreted by the giant out of plane force, namely a very pronounced compressive *σ*_33_(*y*, *z*), acting on the interface under the indenter tip. The compressive out-of-plane component *σ*_33_(*y*, *z*) is localized mainly under the indenter tip with a maximum magnitude of about −8 GPa (Fig. 7) and decreases as a function of the distance from the tip. The maximum *σ*_33_(*y*, *z*) value of −11.4 GPa was observed directly under the indenter in the film loaded with 1.75 N. *σ*_33_(*y*, *z*) stress distribution changes slightly at the FUP/FLP interface and the in-plane width of the *σ*_33_(*y*, *z*) distribution becomes wider and less intensive, especially at the film/substrate interface (Fig. 7). This observation suggests that planar microscopic interfaces between regions of different microstructure and/or residual stress state within monolithic nanocrystalline films contribute to the homogenization of stresses fields and to the decrease of stress concentrations. A comparison of the *σ*_33_(*y*, *z*) distribution from Fig. 7 and the primary crack morphology from Fig. 2 from FUP region shows that the in-plane width of *σ*_33_(*y*, *z*) distribution and the in-plane distance of the two primary cracks (proceeding from the wedge faces to the FUP/FLP interface) correlate. This finding suggests that *σ*_33_(*y*, *z*) distributions inducing high shear stresses on the grain boundaries of TiN thin film in FUP can represent another important contribution to the formation of the primary cracks. Therefore, it can be supposed that the two primary cracks under the indenter in FUP are the result of shear fracture at inter-columnar grain boundaries^41^.

The distribution of the shear stresses *σ*_23_(*y*, *z*) under the indenter tip is presented in Fig. 7, where maximal stresses were observed near the indenter faces with the highest (absolute) value of ~7 GPa. The intensity and gradients of the shear stresses decrease as a function of the distance from the imprint while the stress fields spreads from the indenter edges relatively far outside the indentation zone. The comparison of the imprint morphology from Fig. 2a and *σ*_23_(*y*, *z*) distributions in Fig. 7 shows that the locations of the primary cracks in FLP correlate with shear stress distributions *σ*_23_(*y*, *z*) formed during the indentation. Since in ceramic materials like TiN the shear strength is usually only a fraction of the tensile strength^42^,^28^,^22^, it can be supposed that *σ*_23_(*y*, *z*) distributions contributed also to the formation of two primary cracks in FLP (Fig. 2a).

A comparison of *FWHM*_*δ*_(*y*, *z*) data from Fig. 4 with stress distributions *σ*_22_(*y*, *z*) and *σ*_33_(*y*, *z*) from Fig. 7 allows to interpret the increase in *FWHM*_*δ*_(*y*, *z*) for some *δ* values and sample positions *y* and *z*. The *FWHM*_*δ*_(*y*, *z*) increase observed in Fig. 4 was strongly *δ* dependent (very similar also for TiN 111 Debye-Scherrer rings) and correlated with the occurrence of giant *σ*_22_(*y*, *z*) and *σ*_33_(*y*, *z*) values. This correlation suggests that the changes in *FWHM*_*δ*_(*y*, *z*) can be most likely interpreted by the presence of microscopic elastic strain gradients within various sample regions inside the irradiated sample volume. Also an analysis of the diffraction data (collected after the indenter load was relieved) indicated a relatively weak remaining anisotropic peak broadening, qualitatively similar to that from Fig. 4, which supports the argument of the microscopic strain gradients. A further experimental work is however necessary to understand the effect of the *FWHM*_*δ*_(*y*, *z*) increase in Fig. 4.

### Finite Element Model

**Figure 8:**
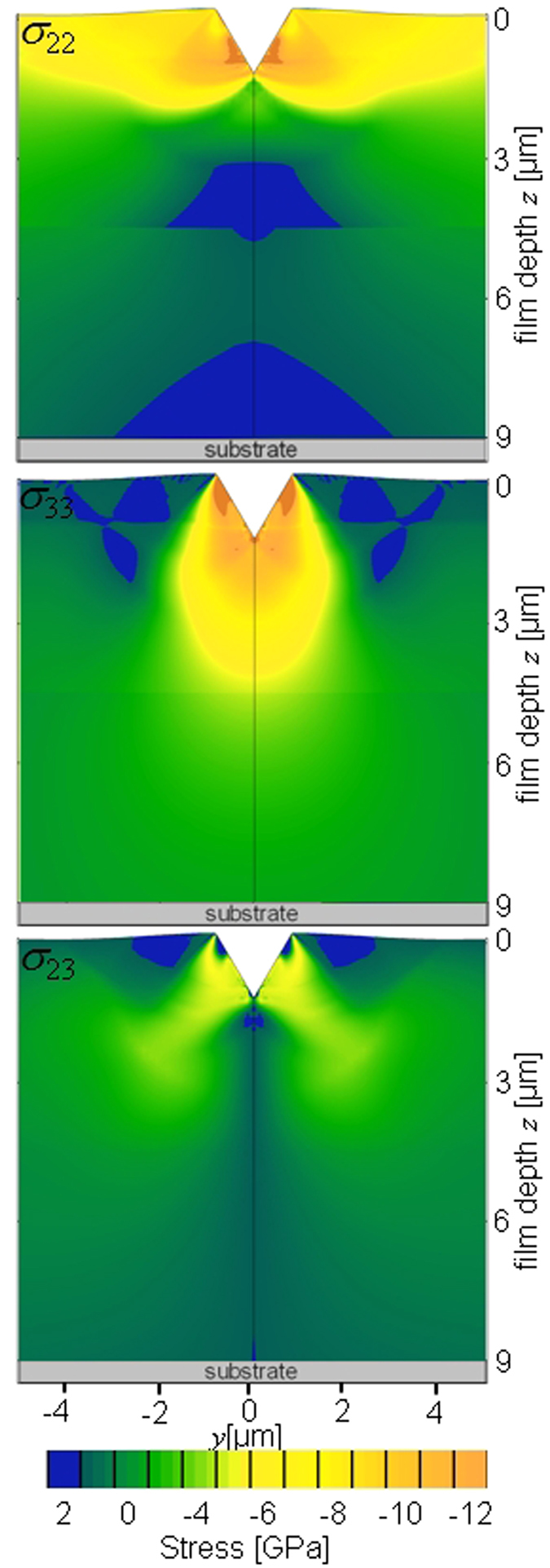
Modelled stress distributions *σ*_22_(*y*, *z*), *σ*_23_(*y*, *z*) and *σ*_33_(*y*, *z*) at the positions *y* and *z* in the 9 μm thick TiN film loaded with 1.4 N. Due to presentation reasons, the actual magnitude of the shear stresses *σ*_33_(*y*, *z*) is shown as −|*σ*_33_(*y*, *z*)|.

Stress changes induced by the wedge indentation in TiN film were evaluated using a FE model. Among the input parameters, residual stress distributions in the unloaded film from Fig. 6 were applied. Other details can be found in the section on Methods. In Fig. 8, modelled cross-sectional stress distributions *σ*_22_(*y*, *z*), *σ*_23_(*y*, *z*) and *σ*_33_(*y*, *z*) are presented. The results indicate a very high in-plane compressive stresses *σ*_22_(*y*, *z*) up to −10 GPa in the film near-surface region next to the indenter, in agreement with the experimental data *σ*_22_(*y*, *z*) from Fig. 7. Also the formation of in-plane tensile stresses under the indenter was verified by the model. Two regions with in-plane tensile stresses (possessing upside down U shape) can be identified in Fig. 8, where the cross-sectional area of the tensile stressed region in FUP is significantly smaller as the one in FLP. The shape of the boundaries between tensile and compressively stressed regions in *σ*_22_(*y*, *z*) distributions (visible in Fig. 8) correlate with the positions of two dominant primary cracks starting at the wedge faces and progressing towards the interface between the film and the substrate in Fig. 2a. In agreement with the experimental observations, also the FE data indicate that the in-plane tensile stress induced by the indenter contributed to the formation of the two dominant symmetrical primary cracks in Fig. 2a.

In the model (Fig. 8), out-of-plane compressive stress *σ*_33_(*y*, *z*) concentrations are localized under the indenter in FUP and the stress concentrations decrease very rapidly as a function of the distance from the indenter (Fig. 8), whereas in FLP the *σ*_33_(*y*, *z*) distribution is very homogeneous. This can be also explained by a different in-plane extent of *σ*_33_(*y*, *z*) distributions along the *y* axis in the model and in the experiment. In the experiment, very localized *σ*_33_(*y*, *z*) concentrations in FUP under the indenter were transferred further into FLP. Both experimental as well as modelled *σ*_33_(*y*, *z*) distributions indicate a formation of tensile out of plane stresses next to the indenter which, besides the very high near-surface *σ*_22_(*y*, *z*) concentrations, could also contribute to the film damage in the near-surface region (Fig. 2a) by initiating intergranular fractures.

In the case of modelled and experimental shear stress distributions *σ*_23_(*y*, *z*) (Figs 7 and 8), both sets of data indicate that the highest shear stresses concentrations are located next to the wedge faces, whereas (i) their magnitude decreases and (ii) their width increases as a function of the distance from the indenter.

In general, the model was able to assess most of the features of the experimentally observed distributions. For a better interpretation, it would be necessary to consider anisotropic film microstructure with columnar grains and nanoscale interfaces.

## Conclusions

A comparison of the fractured TiN thin film morphology after the wedge indentation (Fig. 2a) with the results from the cross-sectional analysis of crystallographic texture (Fig. 3), the diffraction peak broadening (Fig. 4) and the multiaxial stress development (Figs 5, 6, 7) indicates the role of the film microstructure, residual stress gradients and giant stress concentrations induced by the wedge indenter in the crack propagation. The crack propagation within the film was primarily influenced by the specific columnar grain morphology, the presence of two thin film regions with different residual stresses and microstructure, the shape of the wedge indenter and the evolution of the complex cross-sectional multiaxial stress concentrations. Naturally, the crack initiation and fracture were observed at or near the film regions with the highest stress concentrations. It was demonstrated that the presence of microscopic interfaces may result in crack deflection.

Methodologically, it was demonstrated that the novel *in-situ* X-ray nanodiffraction approach offers the capability to quantitatively determine stress distributions as well as texture changes inside an indented thin film volume. Accomplishing this experimentally very difficult milestone opens the way (i) to quantitatively analyse indentation behaviour of nanomaterials with graded microstructure and residual strain, (ii) to quantify stress concentration resulting in various fracture modes, (iii) to design new materials with enhanced mechanical properties and (iv) to adjust finite element models dealing with the material response under the indenter.

## Methods

After the wedge indention, the morphology of the film cross-section was investigated using a Zeiss LEO 1525 scanning electron microscope (SEM). In order to reveal the crack networks across the film thickness, focused ion beam (FIB) milling using an Orsay Physics Cobra Z-05 FIB apparatus attached to a Zeiss Auriga 60 Crossbeam field emission gun (FEG) SEM was performed to prepare a clean cross-section (Fig. 2). The same device was used to machine a free standing micro-cantilever and a sample for *in-situ* indentation presented in supplementary data. The fracture behaviour of the cantilever as well as indenter penetration into the film were observed in a FEG-SEM (Zeiss, LEO 982). The experiments were performed using a wedge diamond tip with an opening angle of 60 degrees mounted on an ASMEC indenter. Maximal load applied on the cantilever was 7.4 mN which corresponds to the fracture stress of 1.4 GPa. In the case of the indent the maximal load was 250 mN.

For the finite element model, plane stress conditions were supposed in the film. For symmetry reasons it was sufficient to model only one half of the lamella with the symmetry condition at the *y*-positon of the indenter tip. The size of the modeled section of the lamella with a width of 30 μm and a height of 200 μm was large enough to avoid boundary effects. The element size ranged from about 0.03 μm in the contact region under the indenter up to about 20 μm in the substrate far from the coating. A total of about 1200 elements with quadratic shape functions and reduced integration (CPE8R) were used and geometric nonlinearity of the analysis was considered. The material behavior used to model the ferritic steel substrate was linear elastic using a Young’s modulus of 210 GPa and a Poisson’s ratio of 0.3. The TiN coating was, for the sake of simplicity, modeled as a monolithic coating of 9 μm in thickness. The texture in the upper part of the coating was neglected. The mechanical behavior of the TiN coating in the FE model was elasto-plastic and a Young’s modulus of 500 GPa and a Poisson’s ratio of 0.21 were applied. The yield limit for the whole TiN film was assumed to be 2 GPa with piece-wise linear strain hardening to 6 GPa after 1% plastic deformation, to 7 GPa after 10% and to 8 GPa after 50% of plastic deformation. The indenter was modelled as a rigid surface. As an input parameter, cross-sectional values of residual stress from Fig. 6 were implemented. The wedge of the indenter had, as in the experiment, an opening angle of 60° and the tip was modeled as perfectly sharp. The contact between the indenter and the film was simulated using the surface-to-surface discretization method of the commercial software package Abaqus in a finite sliding formulation. The perpendicular behavior was hard contact and the tangential behavior was classical Coulomb friction in penalty formulation with a friction coefficient of 0.25. To ensure numerically stable contact conditions in the simulation a small notch of 0.1 μm depth and 0.2 μm width was introduced in the surface of the film at the *y*-position of the indenter tip. The effect of this notch onto the calculated stresses and strains was negligible. During the formation of the indent the nodes in the first 0.8 μm of the symmetry plane directly underneath the indenter tip were allowed to separate from the symmetry plane. On the one hand this avoided excessive large element distortions directly underneath the indenter tip and on the other hand respected possible material separation by means of decohesion while forming the indent. Possible crack formation in the coating was not considered in the simulation.

## Additional Information

**How to cite this article**: Zeilinger, A. *et al.* *In-situ* Observation of Cross-Sectional Microstructural Changes and Stress Distributions in Fracturing TiN Thin Film during Nanoindentation. *Sci. Rep.* **6**, 22670; doi: 10.1038/srep22670 (2016).

## Supplementary Material

Supplementary Information

Supplementary Video 1

Supplementary Video 2
