# Supplementary material for: In-situ Observation of Cross-Sectional Microstructural Changes and Stress Distributions in Fracturing TiN Thin Film during Nanoindentation
Source: Sci Rep. 2016 Mar 7;6:22670. doi: 10.1038/srep22670 (PMC4780078; doi:10.1038/srep22670)
Supplement: Supplementary Information [file srep22670-s1.pdf]

In-situ Observation of Cross-Sectional Microstructural Changes and Stress Distributions in Fracturing TiN Thin Film during Indentation

Angelika Zeilinger, Juraj Todt, Christina Krywka, Martin Müller, Werner Ecker, Bernhard Sartory, Michael Meindlhumer, Mario Stefenelli, Rostislav Daniel, Christian Mitterer and Jozef Keckes

Supplementary Figures:

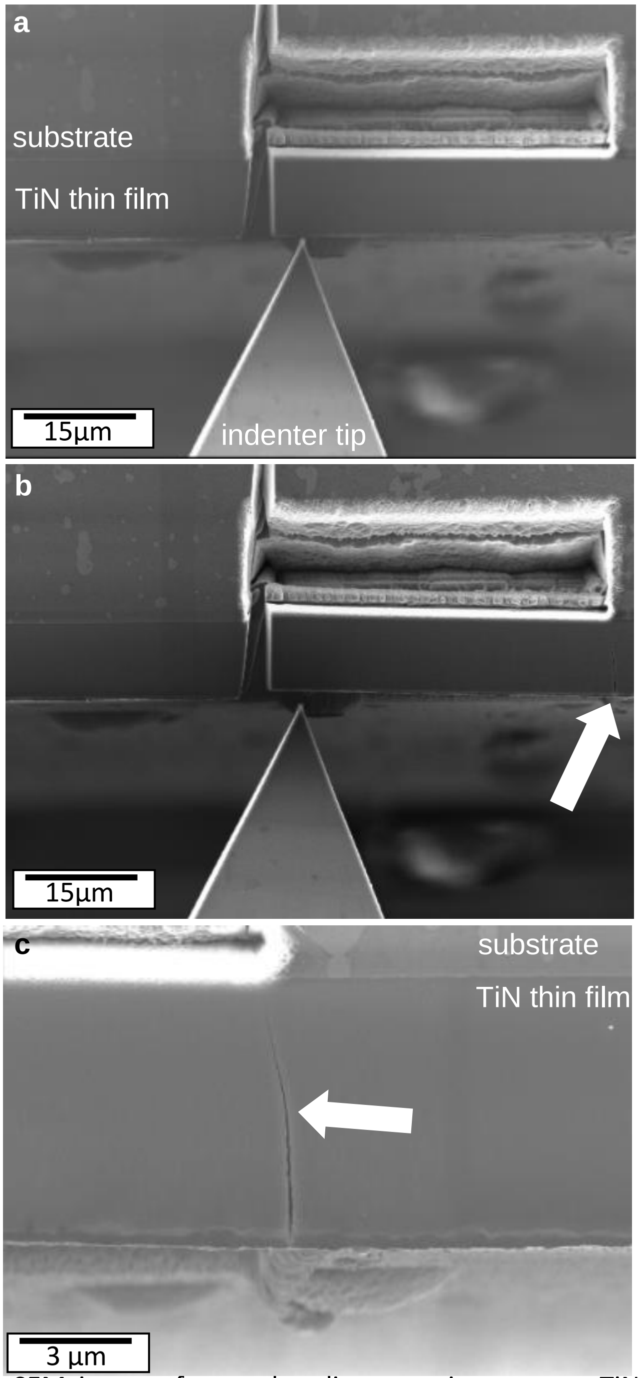

Supplementary Figure 1| SEM images from a bending experiment on a TiN micro-cantilever (*cf.* Video 1) with dimensions of 9 x 9 x 50 µm^3. (a) The cantilever was loaded perpendicular to the TiN thin film surface using a wedge-shaped diamond tip. (b) At the fracture, a crack was formed at the cantilever foot indicated by an arrow. (c) An arrow indicates that the crack path changed at the interface between FUP and FLP, in agreement with the observations performed during and after the in-situ XRD experiment. The corresponding load-displacement curve is presented in supplementary Figure 4.

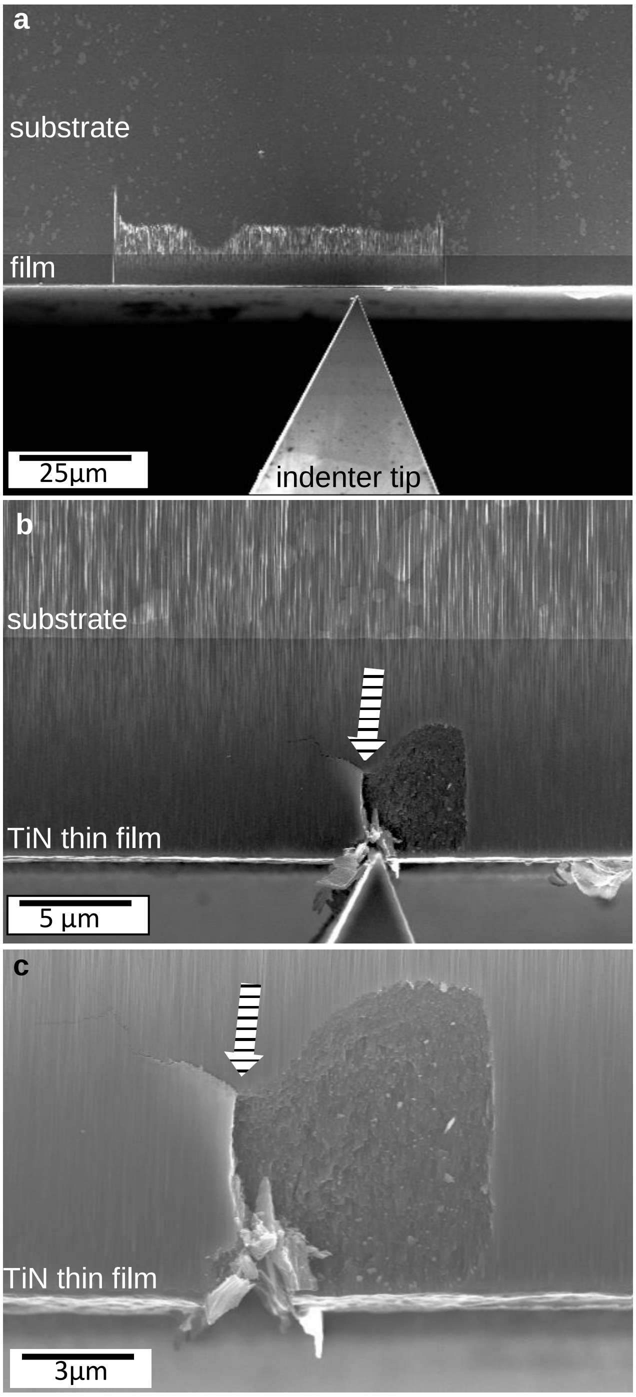

Supplementary Figure 2| SEM images from a wedge indentation experiment on TiN thin film performed with a maximal force of 0.25 N (*cf.* Video 2) on a FIB machined film feature with a width (along the wedge axis) of 30µm. (a) A diamond tip approaching the TiN thin film surface. (b,c) The arrow indicates that at the interface between FUP and FLP, the edges of the crack flakes formed right and left with respect to the indenter axis and the crack growth direction deflected as a result of (i) an abrupt residual stress gradient and (ii) film microstructural variations at this point, as discussed in the article text. Load-displacement curve is presented in supplementary Figure 3.

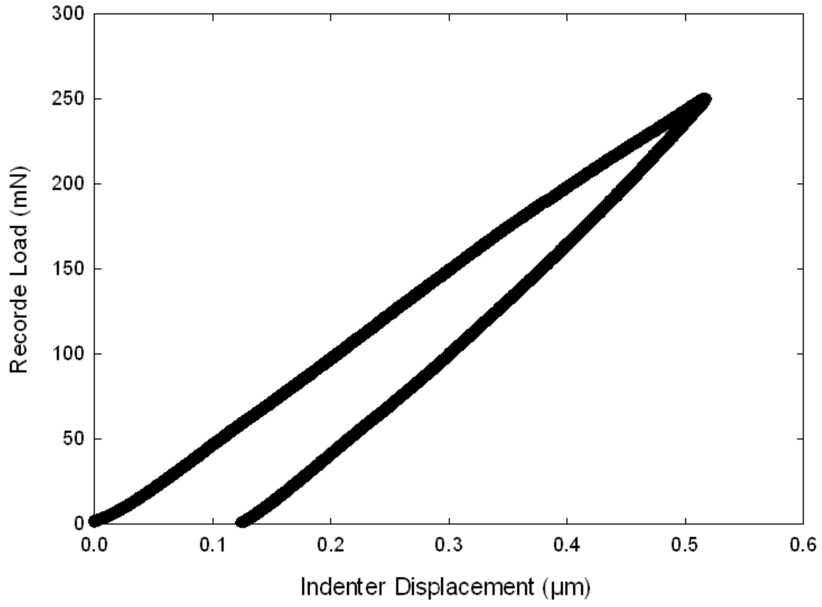

Supplementary Figure 3| Load-displacement curve from a wedge indentation experiment on TiN thin film performed in SEM (cf. supplementary Figure 2).

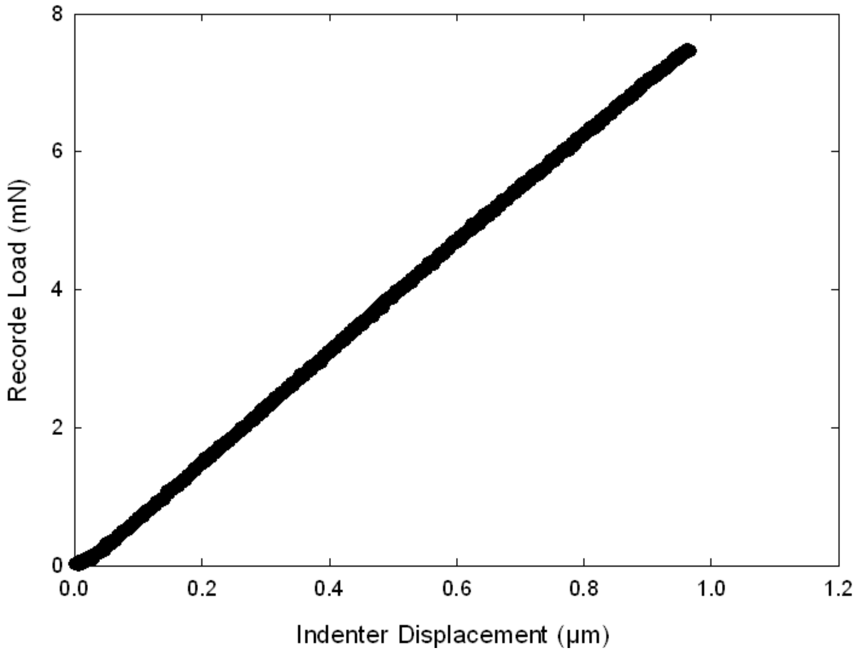

Supplementary Figure 4| Load-displacement curve from a bending experiment on a TiN micro-cantilever performed in SEM (cf. supplementary Figure 1). The almost ideally linear dependence indicates brittle thin film response and an absence of plastic deformation resulting in linear-elastic fracture.

(a)

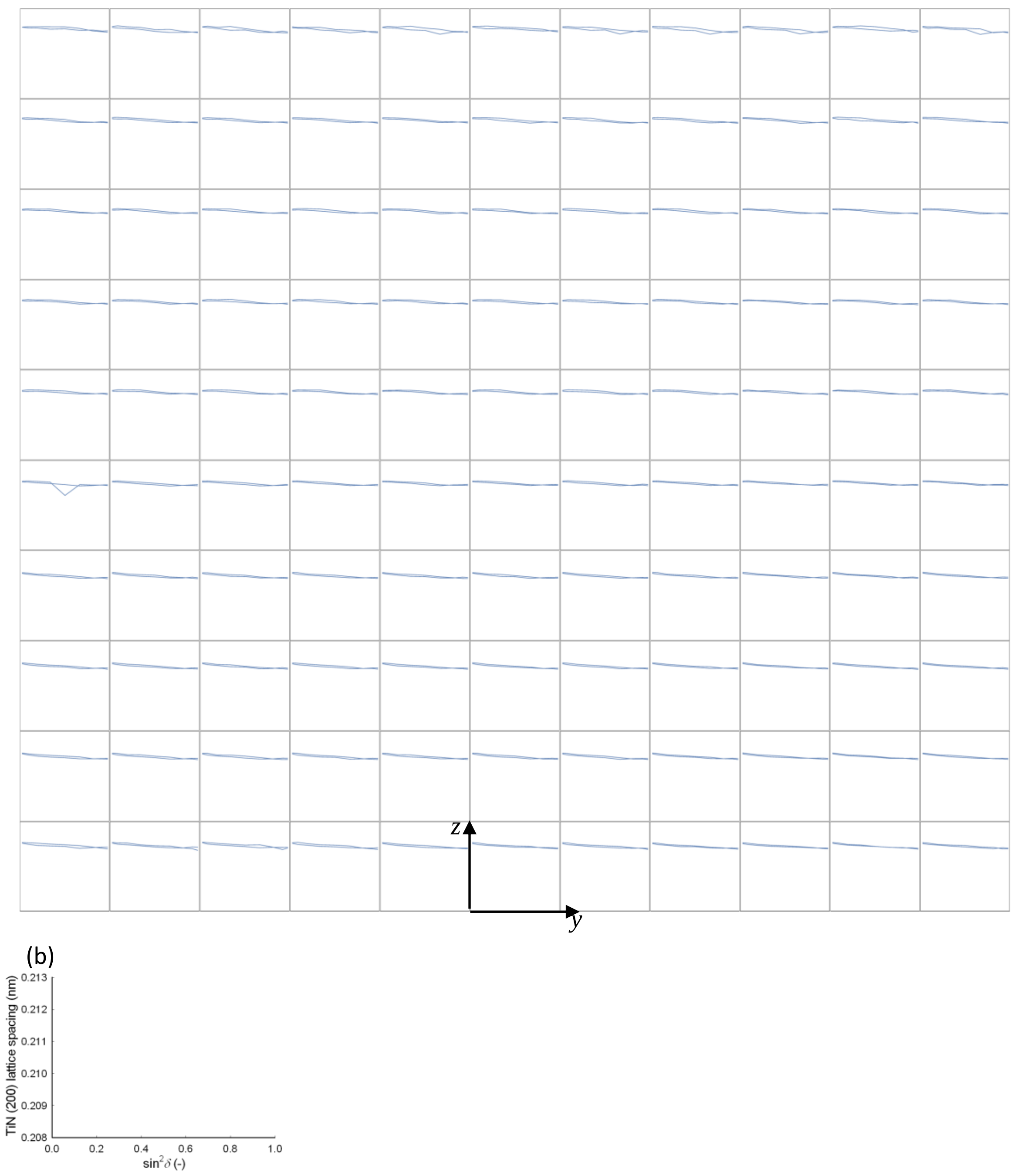

Supplementary Figure 5| Development of TiN (200) lattice spacing $d_{\theta\delta}(y,z)$ for various orientations of the diffraction vector $\overline{Q}_{\theta\delta}$ specified by the angles θ and δ (cf. Fig. 1) and for cross-sectional sample positions y and z. The cross-sectional measurements were performed with a step of ~1 µm along y and z axes. (a) 110 measured dependencies $d_{\theta\delta}(y,z) \sim \sin^2\delta$ document the development of the lattice spacing $d_{\theta\delta}(y,z)$ before the indentation. The 36 measurement points from every $d_{\theta\delta}(y,z) \sim \sin^2\delta$ dependence were used to evaluate stresses in the unloaded sample using Eq. 4. The names and ranges of the vertical and horizontal axes of the individual dependencies in (a) are identical with those in (b) and with those in Fig. 6. The linear dependencies indicate a negligible shear stress $\sigma_{23}(y,z)$ in the unloaded film.

(a)

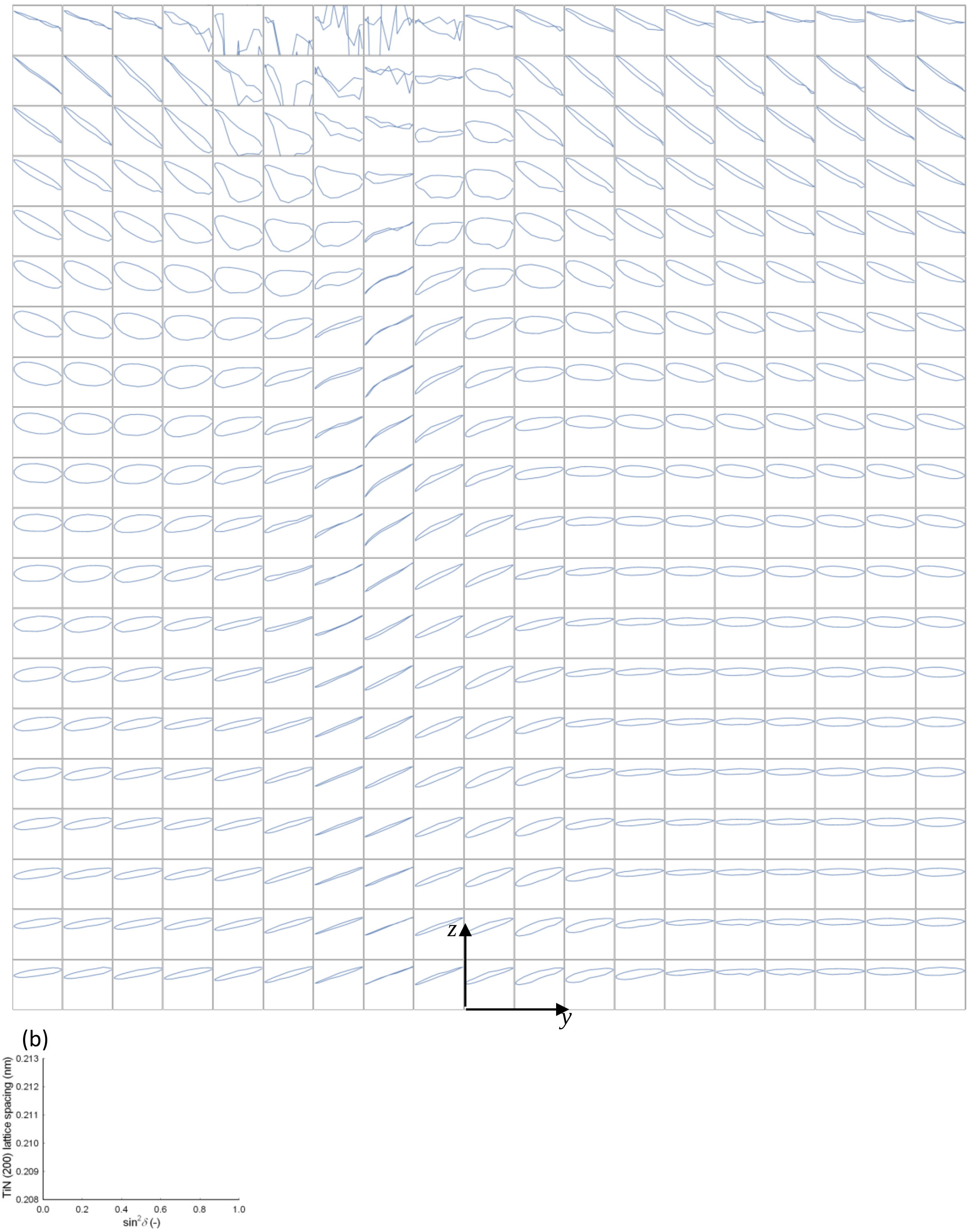

Supplementary Figure 6| Development of TiN (200) lattice spacing $d_{\theta\delta}(y,z)$ for various orientations of the diffraction vector $\overline{Q}_{\theta\delta}$ specified by the angles θ and δ (cf. Fig. 1) and for cross-sectional sample positions *y* and *z*. The cross-sectional measurements were performed with a step of ~0.5µm along *y* and *z* axes. (a) 380 measured dependencies $d_{\theta\delta}(y,z) \sim \sin^2\delta$ document the development of the lattice spacing $d_{\theta\delta}(y,z)$ during the indentation with a load of 1.4 N. The 36 measurement

points from every $d_{\theta\delta}(y,z) \sim \sin^2\delta$ dependence were used to evaluate stresses in the unloaded sample using Eq. 5. The names and ranges of the vertical and horizontal axes of the individual dependencies in (a) are identical with those in (b) and with those in Fig. 5. The split dependencies indicate a presence of shear stress $\sigma_{23}(y,z)$ in the loaded film.

Video Captions

Video 1 A video documenting a bending experiment on a TiN thin film cantilever.

Video 2 A video documenting a wedge indentation experiment on a TiN thin film.
